# New Synthetic Pyridine Derivate as Potential Elicitor in Production of Isoflavonoids and Flavonoids in *Trifolium pratense* L. Suspension Culture

**DOI:** 10.1100/2012/746412

**Published:** 2012-03-12

**Authors:** Marie Kašparová, Tomáš Siatka, Věra Klimešová, Jaroslav Dušek

**Affiliations:** ^1^Department of Pharmacognosy, Faculty of Pharmacy in Hradec Králové, Charles University in Prague, Heyrovského 1203, 500 05 Hradec Králové, Czech Republic; ^2^Department of Inorganic and Organic Chemistry, Faculty of Pharmacy in Hradec Králové, Charles University in Prague, Heyrovského 1203, 500 05 Hradec Králové, Czech Republic

## Abstract

The production of secondary metabolites in *Trifolium pratense* L. suspension culture of the family of legume plants (Fabaceae) is low, and therefore there was an attempt to increase it by elicitation. New synthetic substance, 2-(2-fluoro-6-nitrobenzylsulfanyl)pyridine-4-carbothioamide, was tested as elicitor—a substance that showed the best elicitation effect after 48-hour application of 1 **μ**mol L^−1^ concentration. Maximum contents of genistin (11.60 mg g^−1^ DW), daidzein (8.31 mg g^−1^ DW), and genistein (1.50 mg g^−1^ DW) were recorded, and the production of these isoflavonoids thus significantly increased, when compared with the control, by 152%, 151%, and 400%. The maximum content of flavonoids (5.78 mg g^−1^ DW) and the increase in the production by 142%, when compared with the control, were induced by 6-hour application of 100 **μ**mol L^−1^ concentration. The tested substance showed to be an effective elicitor of phenylpropane metabolism.

## 1. Introduction

Isoflavonoids and flavonoids are, due to their wide spectrum of biological effects, some of the closely studied secondary metabolites of plants [1, 2]. Their major producers are plants of the legume family (Fabaceae). *Trifolium pratense *(red clover) contains especially the following isoflavonoids: daidzein, formononetin, biochanin A, and genistein. The contents of isoflavones were determined in the root, stem, leaf, and bloom of red clover. The highest and nearly equal concentrations were found in the root, stem, and leaf, least of all in bloom [3]. A total of 28 phenolic compounds were identified by high-performance liquid chromatography and mass spectrometry in red clover roots. The most abundant isoflavonoids in pot-grown roots were formononetin glycoside malonate (G-M) (1.51–4.26 mg g^−1^ DW), formononetin (2.21–3.57 mg g^−1^ DW), and biochanin A (1.73–2.17 mg g^−1^ DW), whereas field-grown roots were rich in formononetin-G-M (3.90–4.27 mg g^−1^ DW) and pseudobaptigenin-G-M (1.80–2.58 mg g^−1^ DW). Elevated ozone, cultivation regime, and growth stage affected the levels of phenolics in red clover roots, suggesting sensitivity of root phenolics to biotic and abiotic stress conditions [1].

These isoflavonoids are considered the most important phytoestrogens because of their ability to interact with estrogenic receptors and because of their structural similarity with 17 *β*-estradiol [4, 5]. The red clover extracts are used in female menopause nonhormonal substitution therapy. They decrease the occurrence of menopausal syndromes; have positive effect in the prevention of osteoporosis; reduce the risk of the incidence of cardiovascular diseases and the breast cancer [6–8]. A positive impact of these isoflavonoids in the prostate gland cancer prevention was also noted [9].

*In vitro* methods provide opportunities for propagating and preserving endangered plant species when seed-based methods are not adequate. Tissue culture propagation methods can be used to produce such plants for reintroduction, research, education, display, and commerce. They can also be the basis for tissue banking as a way to preserve genetic diversity when seeds cannot be banked. The number of endangered species that will require *in vitro* methods is estimated to be at least 5,000 worldwide [10]. The plant tissue cultures are a promising source of secondary metabolites. However, their production, when compared with intact plants, is usually lower, and this is the reason why we are looking for different methods and approaches to increase the production. Many traditional strategies can be used to increase the production of secondary metabolites but elicitation is usually one of the most successful.

The principle of elicitation consists in the accumulation of the secondary substances in the plants—a process that is a part of the plant's defense reaction against the influences caused by pathogens or by the plant's environment. The factors that trigger the defensive reactions are called elicitors. Elicitation strengthens the transcription of genes, for instance, phenylpropane metabolism, thus genes that code enzymes which are necessary for synthesis of isoflavonoids and flavonoids (phenylalanine ammonia lyase, chalcone synthase, etc.) [11–13]. The most commonly used elicitors are, for example, fungal homogenates, heavy metals, physical factors, and phytohormones [14–18].

The elicitation method was also used during experiments to influence the *Trifolium pratense *suspension culture production. The best elicitation effects so far have been detected at mercury chloride and at jasmonic acid [18, 19].

Recently, newly synthesized chemical substances have been tested as elicitors in *in vitro* plant cultures. Many of them have proven to possess strong elicitation effect. For instance, fluoro derivates of jasmonic acid [20] and chlorinated pyridine derivates can also be considered suitable chemical induction signals of secondary metabolism of plants [21]. In cases of the *Ononis arvensis*, *Silybum marianum*, and* Genista tinctoria* tissue cultures, synthetic derivates pyrazine-2-carboxamides were observed to be potential elicitors. A more suitable elicitor for the production of isoflavonoids in the callus culture of *Genista tinctoria* proved to be substance B (5-terc-butyl-6-chloro-N-(3-iodo-4-methylphenyl)pyrazine-2-carboxamide). The absolute maximum of genistin production was achieved by a 12-hour action of substance B in a concentration of 2.33 10^−3^ mol L^−1^ (57 times versus control) [22]. Substituted pyrazinecarboxamides markedly influenced production of flavonolignans in *Silybum marianum* callus and suspension cultures. The highest increase in production of flavonoids, by 900%, was detected in the *Ononis arvensis* callus culture [23].

A newly synthesized benzylsulfanylpyridine derivate, 2-(2-fluoro-6-nitrobenzylsulfanyl)pyridine-4-carbothioamide), originally prepared as antimycobacterial or antifungal compound, also influences the plant metabolism—it inhibits photosynthesis in spinach chloroplasts [24]. Therefore, the aim of this study was to prove this compound as a potential elicitor of biosynthesis of isoflavonoids and flavonoids in the *Trifolium pratense *suspension culture.

## 2. Materials and Methods

### 2.1. Instruments

A 200S analytical scales made by Sartorius, Göttingen; a PS 20A autoclave by Chirana, Brno; as HS 31A hot-air sterilizer by Chirana, Brno; a laminar flow workbench by Fatran LF, Žilina; a roller by Vývojove Dílny AV ČR, Praha; a 2010 shaker by Unimax, Heidolph; a CE 1010 spectrophotometer by Cecil instruments, Cambridge; a liquid chromatography (PU-2089 pump, MD-2015 detector, AS-2055 automatic sample injector) by JASCO, Tokyo.

### 2.2. *Trifolium pretense* L. Suspension Culture

A callus culture was derived from the seedling of red clover (tetraploid variety DO-8 obtained from the Breeding Station Domoradice, Czech Republic). The suspension culture was then derived from 1-year old callus culture mechanically by shaking in the Gamborg liquid nutrient medium [25] supplemented with 2 mg L^−1^ of 2,4-dichlorophenoxyacetic acid and 2 mg L^−1^ of 6-benzylaminopurine. The culture was cultivated on a roller at the temperature of 25°C, and a 16hr light/8hr dark photoperiod. The subcultivation interval lasted 14 days. Growth and production characteristics of this culture were determined; an obvious indirect relationship between growth rate and accumulation of secondary metabolites was observed [26].

### 2.3. Elicitation

For elicitation experiments, we used 6-month old suspension culture and the elicitor in the concentrations of 1 *μ*mol L^−1^, 10 *μ*mol L^−1^, and 100 *μ*mol L^−1^ dissolved in 96% ethanol. 1 mL of the elicitor solution was added to each flask (containing 25 mL of the suspension culture) before the end of the exponential growth phase of the culture (on the 21st day of cultivation) [26]. The durations of the elicitor applications were 6, 24, 48, and 168 hours, then the elicited cultures were gathered. 1 mL of 96% ethanol was added to the control cultures. Inspection cultures were collected after 6 and 168 hours since their production does not change notably in such short time intervals. The cells were separated from the media by vacuum filtration, rinsed with distilled water, and dried at the laboratory temperature. The nutrient medium was kept frozen to be used for subsequent analyses. All analyses were carried out in a minimum of three independent samples for each elicitation period and each concentration of elicitor.

### 2.4. Determining the Flavonoids and Isoflavonoids

Spectrophotometric determination of flavonoids was carried out in the extracts of the cells and in the culture media according to the Czech Pharmacopoeia 2009 [27]. The HPLC method with fluorometric detection was used to determine isoflavonoids [28]. A 200 mg sample was extracted twice (in a water bath under reflux condenser) with 10 mL of 80% (v/v) aqueoes methanol for 20 min. The filtrated extracts were combined, and the volume was adjusted with 80% (v/v) aqueoes methanol to 25 mL. After filtration through a 0.45 *μ*m microfilter, the extracts were injected into the HPLC system. Also samples of the culture media were filtrated through a 0.45 *μ*m microfilter and injected into the HPLC system. The HPLC conditions were as follows: a RP-18 Lichrospher column (250 × 4 mm, particles size 5 *μ*m) with a precolumn made of the same material; elution: linear gradient of a mobile phase A (methanol) in phase B (water containing 0.15% (v/v) of phosphoric acid) 30–80% (v/v) from 0 to 9 minutes was followed by the isocratic elution with a mixture of 80% (v/v) of phase A in phase B from 9 to 15 minutes; the flow rate was 1.1 mL min^−1^; the detection was carried out at the 260 nm wavelength. The contents of the monitored substances were quantified by using mathematical method of normalization and by comparing with the calibration curve drawn by the external standard of the same substance. The obtained results were statistically evaluated by the *t*-test at *P* = 0.05.

## 3. Results and Discussion

Successful elicitation depends on many factors that are specific for each elicitor and for each plant tissue culture. This work focused on the elicitor concentration and time duration of elicitor's effect. The results (Table 1) indicate that the elicitor used influenced favorably the production of isoflavonoids in the *Trifolium pratense *suspension culture. The best elicitation effect was observed after 48-hour application of 1 *μ*mol L^−1^ concentration after which maximum amounts of genistin (11.60 mg g^−1^ DW), daidzein (8.31 mg g^−1^ DW), and genistein (1.50 mg g^−1^ DW) were determined. There was statistically significant increase in the production of the above-mentioned isoflavonoids, when compared with control, by 152%, 151%, and 400%, respectively. The positive effect of the elicitation grew with the duration time of the application (6, 24, 48 hours). On the contrary, maximum content of formononetin (7.31 mg g^−1^ DW) was determined after 6-hour application of 10 *μ*mol L^−1^ concentration which represents a 97% increase when compared with control. The stimulation of genistin occurred even after adding the strongest, 100 *μ*mol L^−1^ concentration. Only the production of biochanin A was not influenced favorably. Moreover, the longest (168-hour) application of all elicitor concentrations has decreased the production of isoflavonoids when compared with control.

The production of flavonoids in the elicited *Trifolium pratense* suspension culture (Table 2) was also successful. The best elicitation effect of all elicitor concentrations was recorded after 6 hours. The higher the elicitor concentration, the better the effect observed. The maximum content of flavonoids (5.78 mg g^−1^ DW) was induced by 6-hour application of the strongest, 100 *μ*mol L^−1^ concentration of elicitor, and the increase of production was statistically significant—it increased by 142% when compared with the control. On the contrary, the positive influence of the elicitation was decreasing when the time duration of the elicitor application was prolonged. The longest, 168-hour application of all concentrations of elicitor caused (as in case of isoflavonoids) statistically significant decrease in the flavonoid content when compared with control.

The content of flavonoids and isoflavonoids was not only determined in the extracts of the cells but also in the nutrient media of *Trifolium pratense *suspension cultures. No detectable levels of these substances were found in any of the samples tested. There was thus no secretion of the monitored secondary metabolites into the nutrient medium after the application of this elicitor.

All obtained results indicate that the tested compound, 2-(2-fluoro-6-nitrobenzylsulfanyl)pyridine-4-carbothioamide, has shown to be an effective elicitor of phenylpropane metabolism.

## Figures and Tables

**Table 1:** Production of isoflavonoids in the suspension culture of *Trifolium pratense*.

Elicitor concentration (*μ*mol L^−1^)	Time after elicitation (hours)	Genistin (mg g^−1^ DW)	Daidzein (mg g^−1^ DW)	Genistein (mg g^−1^ DW)	Formononetin (mg g^−1^ DW)	Biochanin A (mg g^−1^ DW)
0 (control)	6	4.61 ± 0.03	3.32 ± 0.08	0.31 ± 0.05	3.69 ± 0.09	0.28 ± 0.04
	24	4.61 ± 0.03	3.32 ± 0.08	0.31 ± 0.05	3.69 ± 0.09	0.28 ± 0.04
	48	4.61 ± 0.03	3.32 ± 0.08	0.31 ± 0.05	3.69 ± 0.09	0.28 ± 0.04
	168	6.30 ± 0.05	3.41 ± 0.06	1.19 ± 0.02	3.91 ± 0.06	0.31 ± 0.02

1	6	7.21* ± 0.09	2.61* ± 0.10	0.52 ± 0.10	4.78* ± 0.10	0.29 ± 0.08
	24	8.89* ± 0.04	5.99* ± 0.09	0.88* ± 0.05	1.12* ± 0.08	0.12 ± 0.12
	48	11.60* ± 0.07	8.31* ± 0.04	1.50* ± 0.06	5.83* ± 0.05	0.21 ± 0.07
	168	1.31* ± 0.12	0.32* ± 0.07	0.23* ± 0.04	0.71* ± 0.04	0.09* ± 0.03

10	6	11.49* ± 0.08	2.98* ± 0.04	1.32* ± 0.08	7.31* ± 0.05	0.28 ± 0.09
	24	7.71* ± 0.10	3.13 ± 0.09	0.31 ± 0.02	6.53* ± 0.09	0.00* ± 0.00
	48	7.39* ± 0.05	2.49* ± 0.06	0.48 ± 0.13	3.62 ± 0.11	0.11* ± 0.02
	168	2.20* ± 0.07	0.62* ± 0.10	0.19* ± 0.05	0.42* ± 0.05	0.11* ± 0.05

100	6	5.11* ± 0.04	2.67* ± 0.15	0.42 ± 0.06	0.59* ± 0.07	0.29 ± 0.07
	24	7.19* ± 0.09	2.92* ± 0.08	0.00* ± 0.00	0.72* ± 0.04	0.19 ± 0.04
	48	8.78* ± 0.11	2.42* ± 0.03	0.14* ± 0.05	2.90* ± 0.06	0.11* ± 0.02
	168	0.67* ± 0.07	0.23* ± 0.05	0.32* ± 0.04	1.89* ± 0.05	0.08* ± 0.12

Levels marked with * are statistically significantly different compared with the control each value represents the mean of three samples ± standard deviation.

**Table 2:** Production of flavonoids in the suspension culture of *Trifolium pratense*.

Elicitor concentration (*μ*mol L^−1^)	Time after elicitation (hours)	Flavonoid contents (mg g^−1^ DW)
	6	2.39 ± 0.07
0 (control)	24	2.39 ± 0.07
	48	2.39 ± 0.07
	168	2.71 ± 0.09

	6	4.87* ± 0.08
1	24	2.67* ± 0.06
	48	2.69* ± 0.03
	168	2.12* ± 0.04

	6	5.05* ± 0.12
10	24	2.72* ± 0.05
	48	2.99* ± 0.02
	168	2.11* ± 0.09

	6	5.78* ± 0.03
100	24	3.56* ± 0.05
	48	2.40 ± 0.05
	168	2.21* ± 0.02

Levels marked with * are statistically significantly different compared with the control. Each value represents the mean of three samples ± standard deviation.
